# Pathways and Prevention for Indecent Images of Children Offending: A Qualitative Study

**DOI:** 10.5964/sotrap.6657

**Published:** 2022-12-02

**Authors:** Alexandra Bailey, Lucy Allen, Eleanor Stevens, Rebekah Dervley, Donald Findlater, Sarah Wefers

**Affiliations:** 1 The Lucy Faithfull Foundation, Epsom, United Kingdom; 2 Goldsmiths, University of London, London, United Kingdom; 3 Centre for Forensic and Family Psychology (CFFP), School of Medicine, University of Nottingham, Nottingham, United Kingdom; 4 Sussex Eating Disorder Service, NHS, Brighton, United Kingdom; 5 Oxleas NHS Foundation Trust, Kent, United Kingdom; University Medical Center Mainz, Mainz, Germany

**Keywords:** Internet, Indecent Images of Children, prevention, pathways, deterrence, sexual offending

## Abstract

The access of indecent images of children (IIOC) is an increasing societal problem; no longer resolvable by law enforcement alone. This research aimed to explore the views of individuals who accessed IIOC, regarding their offending pathways and what they believed could have prevented their offending or encouraged desistance. The qualitative analysis highlights a lack of deterrents for online offending, and the importance of education around legality, consequences, accountability and access to confidential help to prevent online offending. Recommendations regarding IIOC prevention strategies are considered in light of the findings.

## Abstract (plain language summary)

**Background:**

The crime of viewing indecent images of children (IIOC) occurred before the internet, but the online world has made it easier for people to behave illegally. The numbers of people accessing these illegal images has been increasing across the world, and the number of those being detected by the police has continued to grow. Research has so far focused on understanding the types of people that may commit this crime, their motivations for doing so, as well as how likely they might be to re-offend in the future, or commit a contact offence against a child. The understanding of this area of offending has grown steadily over the years.

**Why was this study done?:**

Many involved in preventing online viewing of IIOC recognise that the issue is too big for police to deal with alone. Due to the scale of the problem, criminal justice resources in most countries are not enough to ensure that all those committing this crime are identified, investigated and prosecuted. Focus is now turning to preventing offending in the first place, and deterring potential and continued offenders from viewing IIOC. This study was conducted to understand how people start offending, why they continue, and what might help prevent or deter those who are offending, or are at risk of offending.

**What did the researchers do and find?:**

The researchers interviewed 20 men in the UK who had viewed IIOC online and been investigated by the police. The men were asked about how and why they began to view IIOC, and what triggered and maintained this behaviour. They were asked how their offending could have been prevented, including what initiatives might have been successful in stopping their behaviour if they had known about them before being arrested.

Factors the men highlighted as contributing to their offending included their use of, and relationship with, adult pornography; their initial reaction to viewing IIOC; and the methods used to find the images, including considering the level of risk involved. The men also identified factors they felt continued to facilitate their offending, including sexual gratification and their mind-set at the time of viewing IIOC.

The men identified techniques they had used to try to stop offending, such as willpower, and discussed methods they felt would have been more successful in deterring them from offending. These included receiving information about the illegality and consequences of offending, being aware of available confidential help to stop, and others in their lives being barriers to offending.

**What do these findings mean?:**

These findings suggest that successful prevention strategies for viewing IIOC are possible, and that certain content would make these more effective. This content includes providing messages about the illegality and the consequences of viewing IIOC, and that help is available to stop the behaviour. It also highlighted the importance of prevention targeting for those who might be concerned about another person’s behaviour, and how family and friends could be a protective factor concerning preventing IIOC viewing.

## Highlights

- Adult pornography can act as a ‘*g*ateway’ to initially viewing, and trigger to continued viewing, of IIOC.
- Preventing viewing of IIOC should involve messages that increase awareness of illegality and consequences.
- Availability and visibility of confidential help is important for IIOC deterrence and prevention.
- Bystander awareness and action are additional strategies for preventing IIOC viewing.

In 2019, 59% of the world’s population accessed the internet (Marketing, 2020), revolutionising people’s lives. However, some individuals choose to use technology irresponsibly, including engagement in illegal sexual behaviours (Edward et al., 2011). The perception of anonymity afforded by the internet, combined with the accessibility and affordability of online sexual content (Cooper, 1998) helps create an online context impacting responsible decision-making (Van den Bergh et al., 2008). Demetriou and Silke (2003) argued that the internet itself may trigger individuals to access sexual content, including IIOC.

As access to the internet has increased, a rise in detected online sexual offences has also been noted (Beech et al., 2008; Kloess et al., 2014; Taylor & Quayle, 2003), outstripping current UK law enforcement’s capacity to respond (Sawer, 2017). In 2021, UK police forces had recorded over 100,000 IIOC offences in previous five years (NSPCC, 2021, with the NSPCC previously elevating the estimate of UK-based IIOC offenders to between 450,000 and 590,000; NSPCC, 2016). International law enforcement agencies and EUROPOL have observed a sharp increase in online child sexual offences particularly during the recent COVID-19 pandemic (EUROPOL, 2020). This includes not only IIOC offences, but also live streaming of child sexual abuse, extortion offences, and interactions on dark web child abuse forums. Figures of this nature highlight a need to understand the pathways of IIOC offenders and alternative preventative responses, alongside continued law enforcement strategies. To date, prevention research in this area predominantly focuses on the risk and treatment needs of convicted sexual offenders to prevent re-offending, as opposed to preventing initial offending or offending prior to law enforcement involvement. Therefore, it is important to understand, from those who have committed offences, what might have deterred or interrupted their IIOC related behaviours. The current research aimed to address these points.

Existing research exploring IIOC offender pathways considers existing models based on contact sexual offending, and their applicability to IIOC offenders. Middleton, Elliott, et al. (2006) examined Ward and Siegert’s (2002) pathways model with IIOC offenders and identified similarities in the intimacy deficits and emotional dysregulation pathways, with less support for anti-social cognitions and distorted sexual scripts. However, some research suggests IIOC offenders are more sexually deviant than contact offenders (Babchishin et al., 2015) and cognitive distortions are a prominent component of offence pathways (Kettleborough & Merdian, 2017). Distortions are often related to the distinction between online and offline worlds (Rimer, 2017), perhaps making IIOC-related distortions more offence-specific rather than more global distortions about, for example, children in general (Elliott & Beech, 2009). Some research has typically focused on generalised sexual abuse related distortions, for example use of the Beckett’s (1987) Children and Sex Cognitions Questionnaire in Elliott et al. (2009), perhaps accounting for research discrepancies in offence-supportive thinking.

Research has shown that IIOC offenders differ from contact child sexual abusers not only in terms of their pathways to offending, but also their situations and circumstances. For example, IIOC offenders have less access to children compared to contact offenders (Babchishin et al., 2015). In addition, IIOC offenders tend to have different risk profiles compared to contact offenders, indicated by a low risk of reoffending (Seto et al., 2011). This has led to the development of a specific risk assessment tool for IIOC offenders (Child Pornography Offender Risk Tool – CPORT: Version 2; Eke et al., 2018).

Consequently, research is now starting to examine those who commit IIOC offences independently from those who commit contact offences. Merdian et al. (2020) developed a case formulation model by interviewing 20 men, who had accessed IIOC, about their lives and offending behaviour. Merdian et al. (2020) cited themes around relationship difficulties, early sexual experiences (such as exposure to legal pornography), negative states and dysfunctional coping, including the use of legal pornography as a coping mechanism, in regard to the participants’ background and vulnerabilities. Themes identified around the facilitation of internet offending included viewing the internet as a ‘new world’ and feeling ‘drawn’ into accessing IIOC than rather actively searching from the onset. Themes regarding desistance included enhanced social connection and access to confidential help.

Within the fields of child protection and child sexual abuse (CSA), agencies have called for the adoption of the public health model of prevention (Brown et al., 2011; McMahon & Puett, 1999). Smallbone et al. (2008) set out a Comprehensive Framework for the Prevention of Child Sexual Abuse, by combining elements of the preventative health care model (Goldston, 1987) with Routine Activity theory (Cohen & Felson, 1979). This framework differentiates between primary (targeting whole populations before abuse has occurred), secondary (focussing on ‘at risk’ groups) and tertiary (addressing consequences of abuse to prevent re-occurrence) prevention initiatives. In contrast, the criminal justice model focuses on detecting and prosecuting offenders (McCartan et al., 2018), and predominantly delivers tertiary preventions. Routine Activity theory suggests that an offence can occur providing there is an appropriate target, a motivated offender, and no authority figure or guardian present. It highlights the importance of the potential offender’s environment, and not solely their individual psychological factors. Subsequently, this approach has been influential in the development of situational crime prevention (SCP; Wortley & Smallbone, 2012) for CSA, creating a prevention focus on the environmental factors that facilitate offending.

SCP is underpinned by the idea that offenders will expend the least effort possible when committing crimes (Leclerc et al., 2010). Within the context of abusive sexual behaviour, SCP therefore seeks to prevent abuse, and change the decision–making of potential offenders, through the following measures: increasing risk, increasing effort, removing excuses/reducing permissibility and controlling prompts (Wortley & Smallbone, 2012). SCP is not exclusively concerned with the risk and behaviour of potential or actual offenders; it also recognises the roles of other parties e.g. ‘guardians’ and ‘place managers’ as well as the impact of the environment on offenders’ thinking. This latter point was highlighted in Rimer (2017) who found that those who access IIOC construct boundaries around their internet use which allow them to act in ways they would not in their offline world, driven by feelings of anonymity, and detachment from ‘reality’ when online. Therefore, evidence suggests that SCP is relevant for IIOC offending (Wortley & Smallbone, 2006).

Our understanding of how to deter the initial viewing of IIOC online or to disrupt this behaviour once it emerges, is at an early stage. However, situational efforts are being made to deter individuals from accessing IIOC, for example ‘splash pages’ which make offenders aware of the illegality of the website they are attempting to access (Brennan et al., 2019). In the UK, splash pages are combined with another prevention initiative that removes illegal material from the internet (IWF, 2016), meaning that those who attempt to access indecent images that have been removed instead receive a warning about their attempt to access illegal material. Unfortunately, it is unknown how many times these ‘Splash Pages’ have been accessed, other than anecdotal evidence from those who report having seen them, therefore the success of this initiative in preventing offending behaviour is uncertain.

Additionally, there is pressure on internet service providers to block individuals trying to access IIOC and to reduce their availability (Steel, 2015). This reflects an assumption that technological solutions can resolve issues relating to problematic online behaviour (Carr, 2017); although for some individuals, these strategies will not be sufficient to interrupt their offending behaviour, or to deter them whilst in a state of sexual arousal (Skakoon-Sparling & Milhausen, 2021). Furthermore, the majority of current prevention initiatives offer static information, such a ‘splash pages’, and lack on-going interventions, which may be important for individuals with more entrenched behaviours (Brown & Saied-Tessier, 2015). Therefore, it appears important to include individual psychological factors when deterring potential offenders, which makes the understanding of pathways in light of potential prevention imperative.

The aim of the current research was to understand the journey of those who had committed IIOC offences, their decision making and to consider what might have prevented their behaviour in the first instance or deterred further offending, with a view to informing effective prevention messages and better targeted service provisions.

## Method

### Sample

Semi-structured interviews were conducted with 21 men who were being/had been investigated by police for offences relating to the access of IIOC. All the participants had attended/were attending a community-based psychoeducational programme. The psychoeducational programme is accessed through self-referral and all participants attended the programme on a voluntary basis. The programme is delivered in a group setting with up to ten group members, but can be delivered on a one-to-one basis where appropriate. The programme consists of ten group sessions, or five one-to-one sessions. The contents are based on general sexual offending models, including Finkelhor’s (1984) Precondition Model and the Good Lives Model (Ward, 2002), but adapted to the needs of IIOC offenders. Sessions focus on understanding offending cycles, information on the Criminal Justice System, relationships, the online world, adult pornography use, and relapse prevention.

One participant (008) was removed from the analysis because his investigation ceased due to a lack of evidence. Participants were aged between 32 and 65-years-old, with a mean age of 49.35 years (*SD* = 11.05). Twelve participants had a criminal conviction for possession, making and/or distributing IIOC from the current investigation. Four participants had additional convictions for possession of extreme pornography, fraud, voyeurism and sexual assault. Eight participants had been arrested but not (yet) convicted for the current charges regarding IIOC use. No further descriptive characteristics of the sample such as diagnoses or static risk factors were assessed.

### Materials

The semi-structured interview schedule (see Appendix A in the Supplementary Materials) was developed by the research team in collaboration with an external stakeholder. Questions were selected to explore participants’ use of adult pornography (AP) and first access to IIOC in order to assess their pathways to offending behaviour. Additionally, questions relating to maintenance of offending, ceasing access to IIOC, and their views on potential prevention strategies were included, for the purposes of exploring which prevention strategies may be more effective. Questions were chosen based on their face and content validity to cover participants’ pathways to offending, their offending behaviour and any prevention and intervention strategies they have found helpful or suggestions for additional prevention and intervention approaches. Although the interview schedule was not piloted, iterative changes were made based on discussions amongst the research team and with the external stakeholder until agreement was achieved that the interview schedule was appropriate for the aim of this study.

### Procedure

Prospective participants were sourced from a database of individuals who had been arrested for accessing IIOC and chosen to engage in a psycho-educative programme to address their offending behaviour, ran by The Lucy Faithfull Foundation. Following this programme individuals were given an opportunity to indicate their willingness to participate in research. Those who expressed an interest were contacted via telephone and booked an appointment to meet with a researcher, whereby participants were provided an information sheet and a consent form, prior to participating in an audio recorded interview. Interviews were conducted by a Forensic Psychologist in Training, two Assistant Psychologists and a Practitioner. All interviewers were briefed about the study and discussed questions they had about the interview schedule. No formal training for this interview was given. Participants were verbally debriefed post-interview and provided contact details of relevant support services. The research followed the ethical process of the host organisation and the British Psychological Society. Interview recordings were transferred onto an encrypted laptop and transcribed. Interview times ranged from 38 to 160 minutes. The mean interview time was 87 minutes and 27 seconds (*SD* = 30.85; 30 minutes and 51 seconds).

### Data Analysis

The interview data was analysed using thematic analysis (Braun & Clarke, 2006). Two researchers read the first five transcripts. Each transcript was read through at least twice and notes were made on emerging themes. Both researchers analysed these transcripts to ensure consistency in the identified themes. After these initial transcripts were read, the researchers discussed the emerging themes, exploring any discrepancies and reaching agreement on the identified themes.

The researchers then divided the remaining transcripts equally for analysis. The transcripts were read and notes made in the same method described above. Although no inter-rater reliability was assessed, the researchers met regularly to discuss the data and the emerging themes to ensure consistency and reliability of the coding. A lead researcher provided oversight of the process, to ensure consistency. Quotes were extracted throughout the analysis to ensure that the themes were grounded in the data.

## Results

**Figure 1:**
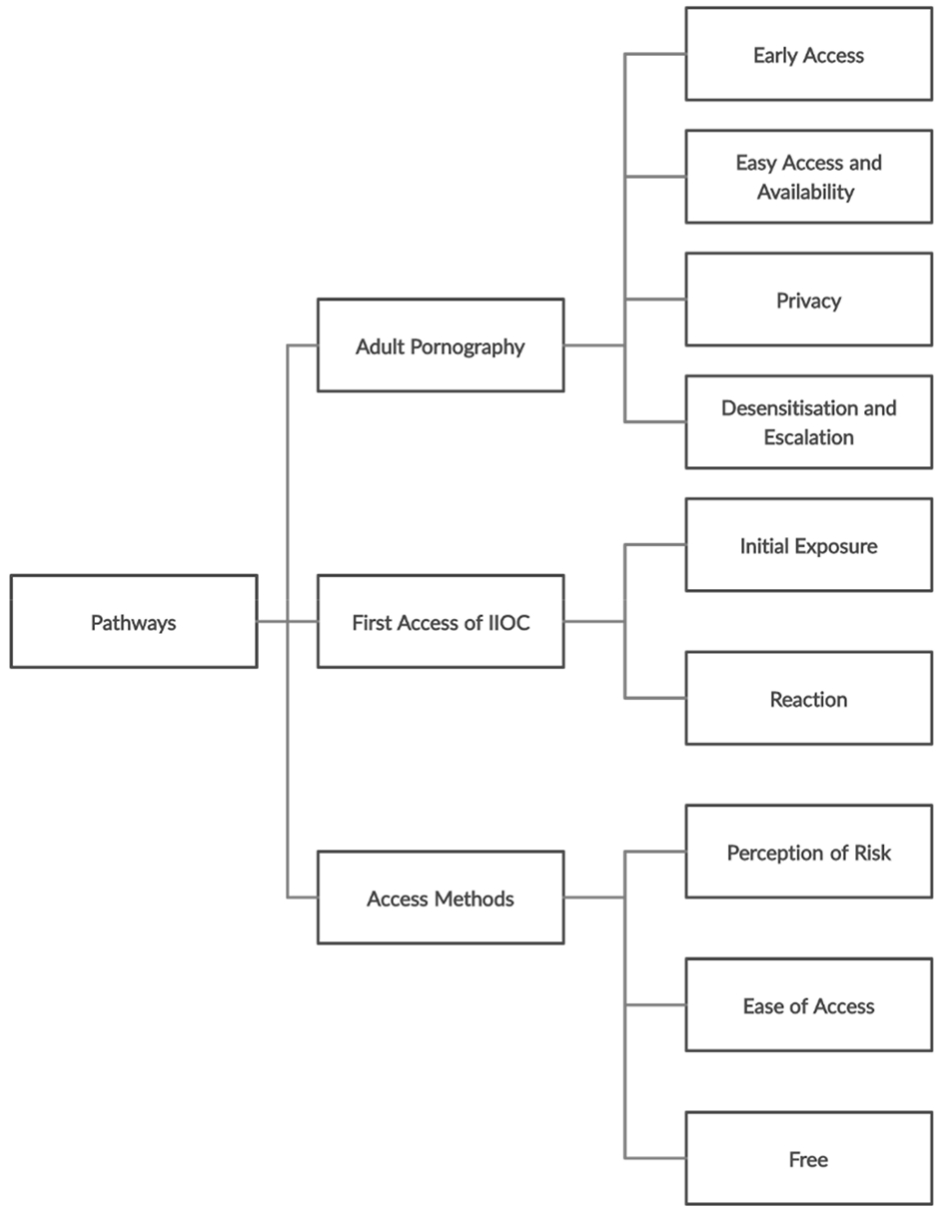
Flow Chart of Themes and Subthemes Relating to the Pathways to Accessing IIOC

**Figure 2:**
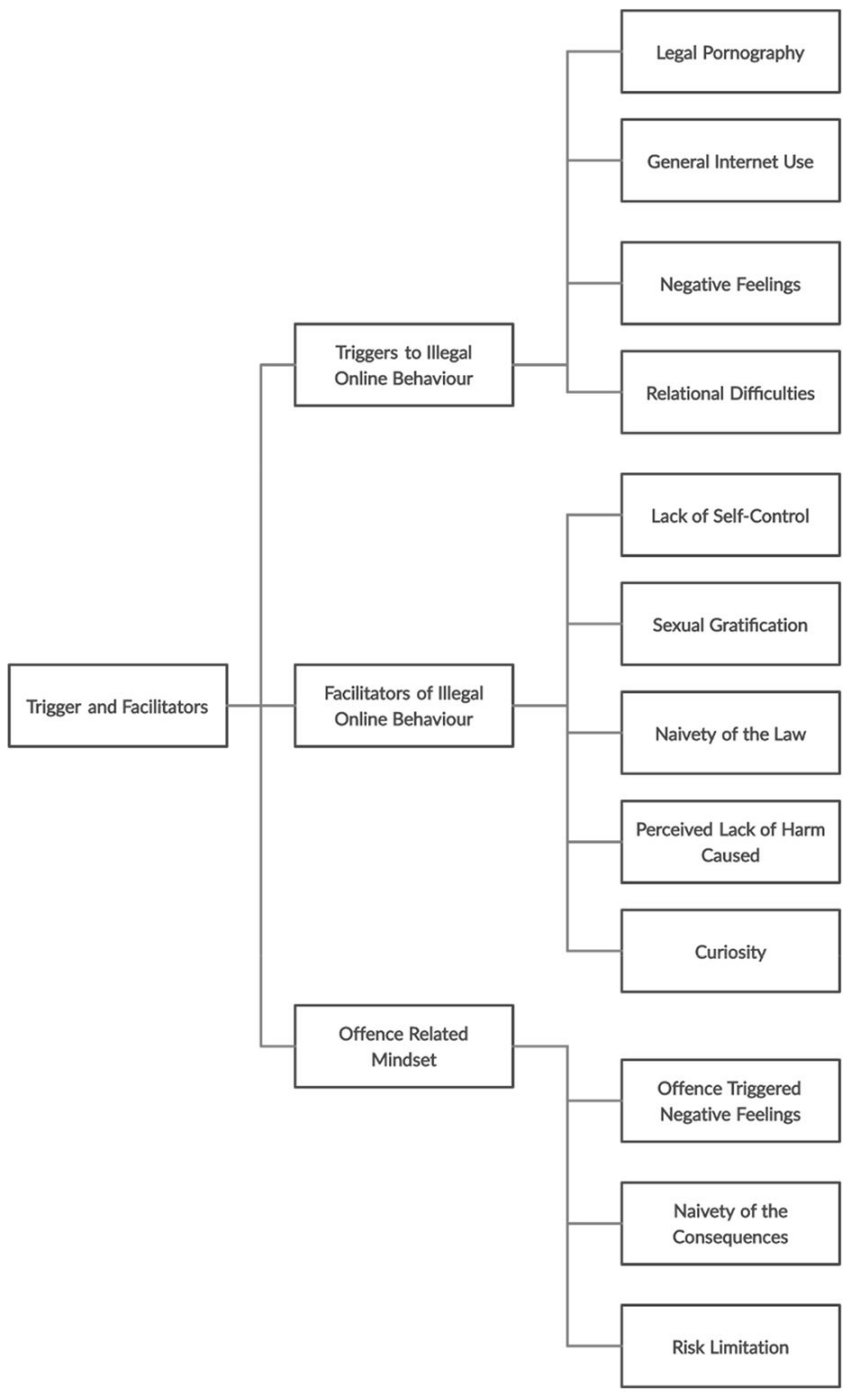
Flow Chart of Themes and Subthemes Relating to the Trigger and Facilitators of Accessing IIOC

**Figure 3:**
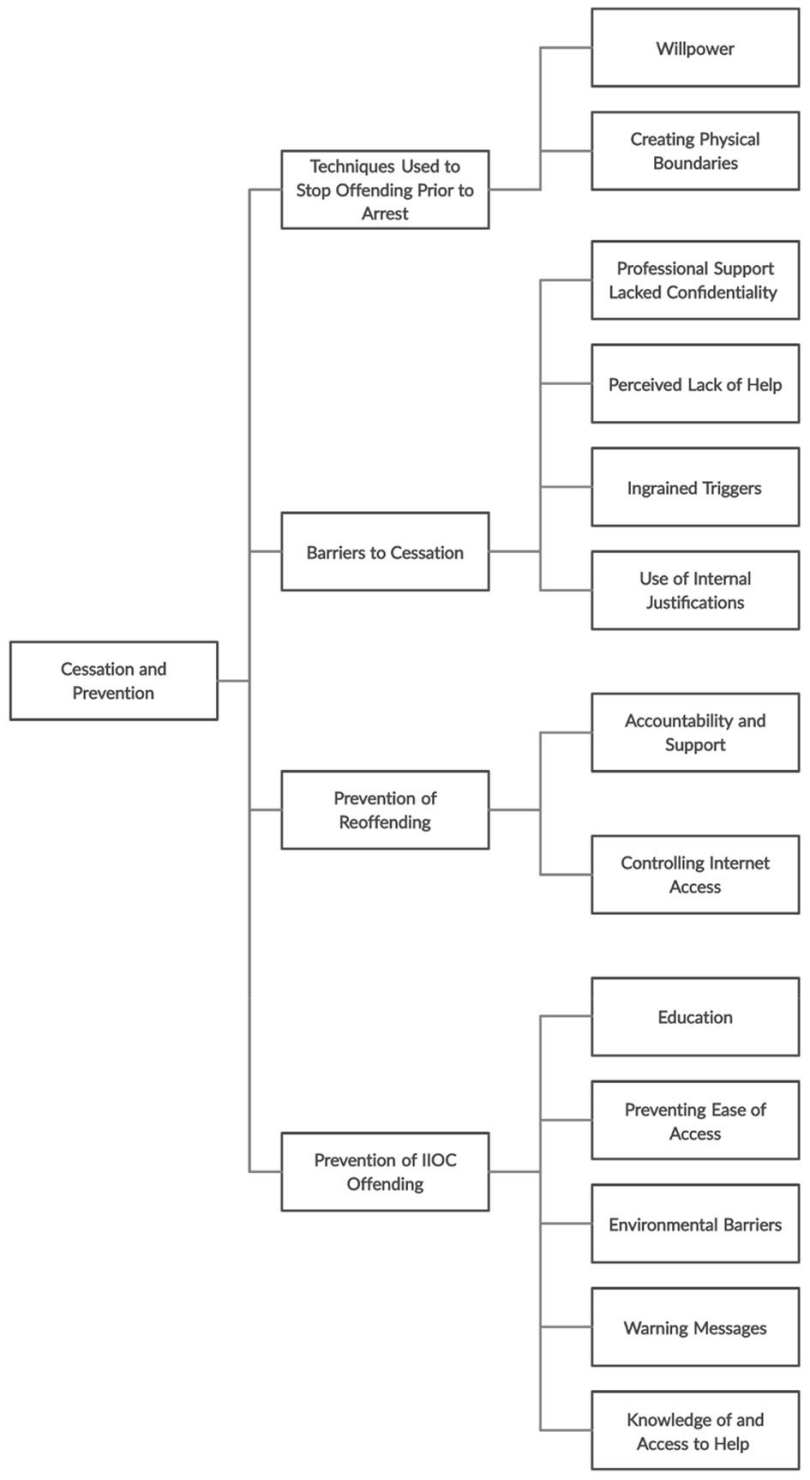
Flow Chart of Themes and Subthemes Relating to Cessation and Prevention of Accessing IIOC

Themes resulting from interviews with all participants have been divided by main areas of interest, including ‘Pathways’, ‘Triggers and facilitators’ and ‘Prevention and cessation’. Each of these focuses are then divided by themes and further subthemes (see Figures 1, 2, and 3). Quotes from participants are included in text, for a complete list of quotes see Appendix B, Supplementary Materials.

### Pathways

#### Adult Pornography (AP)

##### Early Access

Although the age of first AP use was not specifically assessed, half of participants (*n* = 10) volunteered that their first exposure to AP was at an early age: “I reckon I first found it about, about the age of about 10…so that was just finding magazines.” Methods of exposure were linked to participant age; older participants via magazines, younger participants via the internet.

##### Easy Access and Availability Online

Online AP use progressed quickly due to easy access, and most participants (*n* = 15) utilised search engines as AP was available and free. One participant said: “It was just a search. It’s all there isn’t it…you only have to put in some key words and, and the sites will pop up and you just go and look at them.”

##### Privacy

Privacy was paramount, with use typically occurring at home when the participant was alone: “…at night time when all the family were in bed…”

##### Desensitisation and Escalation

Participants identified frequent access to AP contributed to a desensitisation to the type and amount of sexual material needed to achieve similar levels of arousal. The internet enabled access to AP genres participants may not have otherwise accessed; due to inaccessibility, no interest, and/or unawareness of its existence. Eight participants noted becoming ‘bored’ of general AP and pushing boundaries to access more extreme content e.g.: “I got bored with it… [so I started] looking a little bit for variety I guess” and “[The content of the AP I was looking at became] more extreme probably.”. Eighteen participants stated they accessed AP prior to IIOC.

#### First Access of IIOC

##### Initial Exposure

The majority of participants (*n* = 17) stated they accidently accessed IIOC, with nearly half occurring whilst viewing AP (*n* = 8): “Just by accident. I wasn’t looking for it. It just came up on those (AP) sites.” However, four participants described first being exposed to IIOC offline, three of whom went on to intentionally search for IIOC; through search engines (*n* = 2) and talking to others on chat rooms (*n* = 1): “I searched for it.”

##### Reaction

Six participants experienced positive emotional responses to their first exposure to IIOC, for example feelings of: “Excitement, interest, curiosity.” Others experienced negative responses (*n* = 8), including “disgust”. However, some could not remember (*n* = 6) and two experienced both a positive and negative response: “[I felt] shock of the fact that I’ve actually looked at this and that it is so taboo, excitement of having actually done something so illicit and sort of the thrill of that.”

Qualitative exploration of participants’ responses indicated no pattern regarding participants’ decision making, regarding whether they purposefully or accidentally first accessed images, and their initial reaction to IIOC. However, participants acknowledging a primary sexual interest in children (*n* = 4) were more likely to experience positive feelings on initial exposure (*n* = 3).

#### Access Methods

Search engines (*n* = 13), progressive ‘clicking’ (*n* = 9) and file sharing (*n* = 10) were the most reported methods to access IIOC. Less common methods included chatrooms, newsgroups and the ‘dark web’. Methods of choice were influenced by factors noted in the following themes.

##### Perception of Risk

Several participants (*n* = 5) cited their choice of method was based upon perception of risk; if the perceived risk was too high with chances of being detected, the method was avoided. One participant said: “I wouldn’t go on pay sites… I knew there was a greater risk of being caught that way.”

##### Ease of Access

Participants (*n* = 6) stated the ease of access determined the method of obtaining IIOC; with minimal effort being preferable: “It’s the easiest way of doing it… If I’ve had to make too much effort I wouldn’t have ever been doing it.”

##### Free

Ten participants commented on only accessing IIOC that did not cost money; with indications that participants perceived free material as being more acceptable. Participants made statements such as: “I never paid for it.” and “The fact it was there…online and I hadn’t paid for it... It told me it was alright at the time.”

### Triggers and Facilitators

The following themes relate to the factors which participants identified as triggers to the offending behaviour, as well as factors which facilitated the continuation of the offending behaviour.

#### Triggers to Illegal Online Behaviour

##### Legal Pornography

Eight participants cited AP as a trigger to accessing IIOC, typically through progressive ‘clicking’: “…you would start looking at legal pornography… it would always take you back down that route.”. Individuals appeared more likely to be triggered to access IIOC if they felt unstimulated by AP, as highlighted in the ‘Escalation and Desensitisation’ subtheme.

##### General Internet Use

Some participants believed that generally using the internet was a temptation: “As soon as you sit down in front of that PC, the temptation is there.” This was especially apparent if online activities were unstructured, for example: *“*I could be going on there to do something totally different... Just to do normal work and e-mails... And um, you know, then, something would happen and I’d…end up viewing indecent images… [going online is] a potential slippery slope.”

##### Negative Feelings

Participants (*n* = 14) identified negative feelings acting as triggers to accessing IIOC (e.g. boredom, stress etc.), one participant said: “if I’m feeling a bit down or depressed or stressed that would…be a trigger.”. The access of legal and illegal imagery appeared to be a strategy for managing those negative feelings.

##### Relational Difficulties

Participants identified a lack of an intimate relationship, sexual needs not being met and social inadequacy as triggers to accessing IIOC. One participant identified the following factors: “Isolation really…Lack of confidence with the opposite sex, …relationships with the opposite sex, it was sort of no prevention.”

#### Facilitators of Illegal Online Behaviour

##### Lack of Self-Control

Half of participants felt a lack of control over this behaviour and referenced the control they felt the internet had over them. However, participants seemingly exercised control once having reached personal limits regarding what was acceptable content to view, these limits often related to extreme material or younger images. For example: *“*I was powerless over it…I lost control over it…[but] …I never let it progress beyond a certain point.”

##### Sexual Gratification

Ten participants believed sexual gratification facilitated their continued IIOC access; both through arousal at the point of access, as well as meeting needs they felt were otherwise not being met. This included both a deteriorating relationship with sexual partners: “…my physical relationship with my wife was deteriorating all the time… you end up in a situation where you have a preference for masturbation ….”, as well as a sexual attraction to children: “I’ve always been attracted to children.”

##### Naivety of the Law

All participants understood that higher category images were illegal (e.g. those depicting penetrative and non-penetrative sexual activity) however, they reported uncertainty regarding the legality of lower level images (e.g. such as naturist images and images of clothed children). For example: “One thing that didn’t happen until, until I actually got involved with the police was that even images of children that are not undressed but are in provocative poses are illegal.” Further uncertainty involved the age of the child in the image and when this became illegal, one participant said: “At 16 years old… with the naivety of not understanding the internet… there can’t be much wrong in what I’m doing because otherwise it wouldn’t be on screen, they wouldn’t allow it to be there.”. A quarter of participants continued to view certain images as they lacked understanding of their illegality. One participant was previously so confident that the images he viewed were legal, that he was initially unconcerned by his arrest.

##### Perceived Lack of Harm Caused

Participants (*n* = 5) continued to access IIOC as they believed they were causing no harm to the children in the images or anyone else. Various justifications and cognitive distortions were used to distance themselves from any harm caused to victims, such as: “as far as I was concerned as long as they looked to be enjoying it, then that was fine.”

##### Curiosity

Six participants said curiosity facilitated their continued access of IIOC, often related to excitement and a challenge to see what they could find: “Curiosity… Just kept leading me astray… I guess, to see just, to see what else was there.”

#### Offence-Related Mindset

##### Offence Triggered Negative Feelings

Participants talked about experiencing negative feelings (e.g. guilt, shame) immediately after or sometime after accessing IIOC: “Guilty. Terrible. As I said, broke my computer...” A proportion of participants expressed awareness of the immoral and illegal aspect of viewing IIOC, and reflected that children were not sexual objects. However, whilst accessing IIOC, participants pushed thoughts of morality and illegality aside, for example: “So I had all the guilt and everything else like that but during it, it was ok. It was my thing. I wasn’t hurting anyone.”. Some participants reported this was facilitated by feelings of excitement and arousal: “Knowing I was doing wrong and there was a risk of being caught. But the excitement overcame that.”

Participants also identified that negative feelings and detection concerns were more pronounced at the beginning of their offending, with concerns lessening the longer they offended without consequence: “… if you’ve been offending a long time… your barriers just break down.”

##### Naivety of the Consequences

Some participants were aware of legal and personal consequences (e.g. being arrested, losing relationships and jobs) but their understanding of the actual impact of these was less present (e.g. no participant considered the Sex Offenders Register, prison, Disclosure and Barring Service checks etc.), e.g.: “[I didn’t consider] the fact that I might end up on a sexual register of some sort.”. Consequences were also considered from a personal view point (how it would impact themselves) rather than how it would impact others: “I [didn’t consider] the full effect this has had on my wife…and… my son…they are probably the two things that since my arrest really… hit home the extent of [the consequences] …”

##### Risk Limitation

The risk of being caught by a family member was deemed more controllable (e.g. by using a separate laptop, clearing browser history, offending whilst others were asleep or out), yet lengths to reduce police attention were also taken (e.g. deleting material, destroying hard drives): “…I purged many times and you know replaced discs and drilled holes in them…”

### Cessation and Prevention

Most participants (*n* = 19) stopped accessing IIOC due to law enforcement intervention; only one ceased their behaviour prior to their arrest. However, 17 participants had tried to stop their behaviour prior to arrest, for example: “…it would be just after viewing images…, I would close everything down and…say, that’s it…that was awful. You are a fool…We’re going to stop that right now…”. A number of reasons for this were identified including a fear of detection, and negative views of their own behaviour: “Fear of getting caught. There’d been something on the TV or news or what have you which was of people getting caught…”

Participants who did not attempt to cease offending appeared to have accepted the justifications they were using, including: “I had convinced myself there was no harm to it.”, and felt the desire to access outweighed the desire to stop: “The urge was too strong…The desire…couldn’t help myself.”

#### Techniques Used to Stop Offending Prior to Arrest

Cessation attempts were mostly self-imposed; only one participant disclosed to another person about their problematic use of online pornography and requested the installation of filtering software.

##### Willpower

Participants attempted to stop offending by being ‘strong’, e.g.: “It was willpower.”

##### Creating Boundaries

Participants created physical boundaries between themselves and IIOC, e.g. “Deleting all the stuff on the computer… burning a hard drive, melting a hard drive…”

#### Barriers to Cessation

##### Professional Support Lacked Confidentiality

Participants generally believed disclosure to professionals would result in arrest; a lack of anonymous help therefore became a barrier to seeking help pre-arrest, e.g. “…because [disclosing offending] of course meant confessing and of course… in this country you have reporting law…”

##### Perceived Lack of Help

Participants felt they were unable to address the underlying reasons for their offending behaviour, either due to how they felt about their behaviour or believing there was nowhere to access help. One participant said: “I think I thought there wasn’t any out there and I was too ashamed… I was so worried about the consequences that I just shut mind down to it.”

##### Ingrained Triggers

Due to a lack of intervention, the triggers that influenced the onset of offending remained present, which then contributed to relapses. These included emotional triggers, e.g. “…something may have happened. Some incident or it could be anything… I’d drop back down into a bad mood again…”, and becoming desensitized to legal material: “…when I again got the re-interest and got bored of the adult porn.”

##### Use of Internal Justifications

Some participant also cited that the justifications utilised in maintaining their offending behaviour also contributed to relapses: e.g.: “I just tried to force it into places it fit my conscience…, so, if I wasn’t looking at adult involvement…that was kind of more ok.”

#### Prevention of Re-Offending

The following themes are factors which participants identified as important to the prevention of their own reoffending following arrest, including methods they had in place at the point of interview.

##### Accountability and Support

Participants’ urges to reoffend were managed by the knowledge that people were monitoring them, and therefore acting on their urges was deemed too risky. Accountability also related to external sources of support utilised post-arrest, with six participants citing counsellors, courses and support from partner/friends: “I think accountability is very important… having quite a lot of people who know means that there are more people… that can keep me accountable...” Additionally, participants identified both the emotional and practical support of loved ones (“…she’s still supportive as much as she can. I think it’s a key thing in this, isn’t it, to feel accepted and not to be judged.”), as well as feeling a necessity to rebuild the trust of loved ones were motivators to not reoffend (“I’ve been given a chance from them and I have to work very hard to keep, to keep up my end of the bargain on it.”).

##### Controlling Internet Access

Following arrest, external sources became involved in controlling the participants’ internet access (installing monitoring software; supervising internet access and setting passwords; structuring time) instead of them relying on willpower and self-enforced boundaries. One participant said: “My wife and I have put on [parental control software] which… allows her to look what…I’ve been viewing and it blocks dubious websites…”

#### Prevention of IIOC Offending

The majority of participants reported never encountering online or offline deterrence messages (*n* = 14, *n* = 15). Those who did encounter deterrence messages did not find these to be effective. For example, with online messages participants resolved these barriers by closing webpages, clicking the back button and ignoring warnings, e.g. “I do remember some messages saying you may be accessing illegal images… I carried on.” Participants who encountered offline deterrence referred to their own thoughts about their behaviour, faith and news articles featuring arrests, e.g. “…stories on the news about people being caught…”. Thereby, highlighting the lack of prevention messages experienced by participants.

##### Education

Over half (*n* = 11) suggested the lack of education as a primary prevention focus for young people and society, e.g.: “Education is one of the strongest weapons… I think that the problem has to be tackled, at a much earlier stage.”

##### Preventing Ease of Access

Participants suggested that an effective form of online deterrence involved preventing ease of access to IIOC, through the active removal of illegal material as well as online barriers to accessing material. Eleven participants explored the contribution that Internet Service Providers, search engines and web companies could make in order to prevent IIOC access: “It’s the service providers and Google working harder to block, block results or something.”

Additionally, seven participants discussed making IIOC less available online: “What I want to happen is that child pornography becomes… something that you have to actively search for… Take all sorts of extraordinary steps in order to access it… it’s too easy.”

##### Environmental Barriers

Eight participants reported environmental barriers, i.e. the presence of others, were effective in preventing them from accessing IIOC: “Large barriers is…when you live in a house with other people. It’s having other people around.”

##### Warning Messages

Participants felt that online and offline messages regarding the illegality of IIOC use (*n* = 4), e.g. “More education around the law.” and warnings about their access (*n* = 7) and the consequences of viewing IIOC (*n* = 6) were techniques participants believed would have prevented their use of IIOC: “…if the warning was good enough like saying, we can know, we know who you, we know where… We know where the search is coming from…” and “ISPs sent letters saying you appear to have accessed.”

##### Knowledge of and Access to Help

Participants stated that having knowledge of what help was available (*n* = 5) and knowing how to access this would have discouraged them from accessing IIOC. For example, “…if I was able to access a helpline or help group…” and “…that would be the key thing if… I’d… known… that there was… proper help and assistance…” and “… other European countries… have adverts on the TV…, encouraging people to go and get help… we need something like that that’s gonna have a big impact… it’s showing that the government are doing something to try and battle this problem.”

## Discussion

The current research aimed to explore offending pathways of IIOC offenders and to consider these in light of potential prevention strategies for this type of offending. Semi-structured interviews were conducted with individuals arrested for accessing IIOC. This research enhances current literature regarding the understanding of psychological and situational factors in deterring those at risk of accessing IIOC.

The current findings relate to existing models of child sexual offending. In line with Ward and Siegert’s (2002) Pathways Model, participants identified relationship issues (intimacy deficits), early exposure to pornography and sexual gratification (deviant sexual scripts), negative feelings and lack of self-control (emotional dysregulation), and internal justifications (antisocial cognitions) as important contributing factors to their offending. The current findings therefore indicate that the Pathways Model may also be applicable to IIOC offending. In addition, the present study highlights that situational crime prevention (SCP; Wortley & Smallbone, 2012) is not only relevant to contact sexual offending but to IIOC offending as well. Environmental factors and the make-up of the online landscape were discussed as facilitating factors for IIOC offending and also promising avenues for prevention efforts.

The research highlighted the key role of AP, both as a ‘gateway’ to viewing IIOC in the first instance, but also a potential trigger for continued viewing due to a lack of boundaries online between legal and illegal sexual material. Participants highlighted the potential of ‘accidental’ initial access to IIOC through AP, validating the lack of purposeful initial exposure reported in previous research (Merdian et al., 2020). On a secondary prevention level, this finding might indicate that behaviours could be targeted and interrupted for those at risk, within an AP space. However, the research suggested that an attempt to prevent offending during access to sexual material would unlikely be effective, as consequences or negative thoughts about the behaviour would be ignored when experiencing arousal. This appears to further validate the concept of impairment to responsible decision making at times of arousal (Van den Bergh et al., 2008), therefore prevention initiatives should be visible to individuals when they are not currently experiencing sexual arousal.

The current research supported elements in the pathways that had been cited in previous research, including the presence of justifications and distortions (Kettleborough & Merdian, 2017). The distortions participants cited appeared offence-level (Elliott & Beech, 2009) and seemed focussed around key issues, including the harm caused to victims in the images, as well as the participants own sense of responsibility and seriousness of their behaviour. The role of these distortions seem to relate to the maintenance of a behaviour that had already commenced, but also to justify relapse. However, at times it appeared that participants’ where unaware of distortions they were voicing, indicating for some a continued protective function even after the cessation of behaviour. Distorted thoughts would therefore require challenging within any deterrence initiative; namely the harm caused, offence severity, and the empowerment to control and stop the behaviour.

An additional theme was around perception of risk. This related to methods participants used to engage in offending behaviour and thinking patterns regarding whether detection was a potential outcome. Participants clearly stated that receiving messages indicating detection as a real possibility, with exact consequences highlighted, would have been an effective deterrent for them. Such warning messages may be an effective secondary and tertiary prevention effort. By increasing awareness of the legal and personal consequences to offending, it could be hypothesised that an offender’s perceptions of risk might be heightened, as well as removing common excuses for the behaviour that centre around its legality. This is congruent with central aspects of SCP (Wortley & Smallbone, 2012), and could impact offender thinking patterns, such as the detachment from ‘reality’ (Rimer, 2017).

This research also highlighted the need for messages to be reinforced in dynamic and visual ways. This could indicate that previous initiatives, such as ‘splash pages’, might not present the information in the most effective manner. Therefore, secondary and tertiary prevention and deterrence initiatives need to be more visual and interactive as this will more likely demand the attention of the viewer, rather than a static message that participants reported often ‘clicking off’.

It was also evident within the research that signposting individuals to continued sources of anonymous and confidential support was an important aspect of the prevention process. Providing on-going intervention appears to have a beneficial effect on retention and understanding (Brown & Saied-Tessier, 2015). Participants indicated that their offending behaviour was linked to psychological and relational issues, such as relationship breakdowns and difficulties coping with negative feelings (Merdian et al., 2020), indicating that for some individuals the situational deterrents would be insufficient alone, with further access to confidential support being preferential.

In addition, reflective of the concepts of SCP (Wortley & Smallbone, 2012) and Routine Activity theory (Cohen & Felson, 1979) the research highlighted the importance of accountability and protective others in the prevention of online offending. Participants’ identified that self-imposed attempts to stop were unsuccessful, but knowing others would be acting as guardian or ‘watching’ them (Rimer, 2017) was more helpful, contributing further validation for targeting IIOC offenders using a public health model approach (Brown et al., 2011), for example through education around illegal online behaviour targeting those who may offend but also their family members, friends and housemates.

### Limitations and Future Research

Individuals who have accessed IIOC are a heterogeneous group with varying backgrounds and motivations (Elliott & Beech, 2009). This study utilised a small sample size and it is unclear whether similar pathways and desistance messages would be relevant with different typologies of offenders. For instance, it is unknown whether those driven by an exclusive interest in images of pre-pubescent children would be deterred in the same way as those who have an interest in images of adolescents, those with a non-exclusive sexual interest in children or whether it would be effective with those who are involved with online solicitation and grooming. Also, the study only focused on those who had been detected by law enforcement; those avoiding detection may require different prevention messages. Future research analysing different types of online offenders may lead to more effective deterrence strategies.

Additionally, this sample had approached the community programme voluntarily. Therefore, they may not be reflective of those with more extreme cognitive distortions, who may be less likely to seek professional support. Furthermore, due to the voluntary and self-report nature of both the programme and research we are unable to verify each participant’s online behaviour. Subsequently, we cannot be sure the participants were honest about their online behaviour.

However, the results indicated that prevention and deterrence could be possible with the right messages (such as those around legality and consequences) at the right time (such as when not engaged in sexual behaviour online). Although, there was also indication that this alone may not be enough and individuals required a source of accountability, such as through family and friends or the ability to seek professional help without fear of law enforcement involvement. This shows that initiatives to deter this behaviour are best done in conjunction with the ability to seek help and accountability.

In summary, a successful initiative would need to target a number of areas; removing excuses and increasing the perceived risk and consequences for those who might view IIOC, providing an outlet to seek confidential help for their behaviour, and targeting those who could offer accountability and encouraging them to notice changes or concerns in someone else’s online behaviour. The results also indicate that certain kinds of resources in relation to CSA prevention may have wide levels of take-up if they draw on findings from the narratives of those who have engaged in the offending behaviour about effective prevention strategies. However, these narratives were retrospective in nature and it would be imperative for further research to explore whether these strategies prevent offending in the first instance, or discourage continued access, on a long-lasting basis.

## Supplementary Materials

The Supplementary Materials contain the online appendices for this article (for access see Index of Supplementary Materials below). Appendix A is the interview schedule used for this study. Appendix B lists quotes per theme identified for this study.



BaileyA.
AllenL.
StevensE.
DervleyR.
FindlaterD.
WefersS.
 (2022). Supplementary materials to "Pathways and prevention for indecent images of children offending: A qualitative study"
[Appendices]. PsychOpen. 10.23668/psycharchives.8411
PMC1178943539902159

## Data Availability

The data that support the findings of this study are available from the corresponding author, AB, upon reasonable request.
